# A method for parallel microscale protein labeling and precise control over the average degree of labeling (aDoL)

**DOI:** 10.1038/s41598-023-36163-8

**Published:** 2023-06-02

**Authors:** Qiaoqiao Ruan, Cheng Zhao

**Affiliations:** Applied Research and Technology, Abbott Diagnostics Division, AP-20, Abbott Laboratories, 100 Abbott Park Road, Abbott Park, IL 60064-6016 USA

**Keywords:** Biochemistry, Biological techniques, Biophysics

## Abstract

A widely used approach for protein conjugation is through the lysine residues reacting with NHS- or other active esters. However, it is a challenge to precisely control the degree of labeling (DoL) due to the instability of active ester and variability of reaction efficiencies. Here, we provide a protocol for better control of aDoL using existing Copper-free Click Chemistry reagents. It is a two-step reaction with one purification in between. Briefly, proteins of interest were first activated with azide-NHS. After removing unreacted azide-NHS, the protein-N_3_ is then reacted with a limited amount of complementary click tag. Our studies have shown the click tag will fully react with the protein-N_3_ after 24 h’ incubation, and therefore does not require additional purification steps. As such, the aDoL is equal to the input molar ratio of the click tag and the protein. Furthermore, this approach offers a much simpler and more economical way to perform parallel microscale labeling. Once a protein is pre-activated with N_3_-NHS, any fluorophore or molecule with the complementary click tag can be attached to the protein by mixing the two ingredients. Quantities of the protein used in the click reaction can be at any desired amount. In one example, we labeled an antibody in parallel with 9 different fluorophores using a total of 0.5 mg of antibody. In another example, we labeled Ab with targeted aDoL value from 2 to 8. In a stability comparison study, we have found the conjugated fluorophore using the suggested click protocol stayed attached to the protein longer than with standard NHS-fluorophore labeling.

## Introduction

Proteins play a central role in biology and making them visible or detectable is essential for researchers to study their functions and interactions. In 2001, Sharpless published a landmark paper describing a simple strategy to couple two molecules together by “clicking”^1^. Later, Meldal and Bertozzi further developed the chemistry and made it available for broad application^2,3^, for which 20 years later, their works were recognized and awarded Nobel Prize in chemistry. The bioorthogonal chemistry or click chemistry, has demonstrated great advantages for targeting, with high specificity, modified individual proteins with probes or tags in complex systems^4–10^. There have also been other significant advancements in chemo- and regioselective labeling methods to achieve a single tag attached to native proteins^11,12^. For comprehensive reviews, and books on protein bioconjugation, please see^13–15^. It is also worth mentioning that the cysteine residue is another popular target for site-specific protein conjugation. Most proteins do not contain native free thiols that can be targeted, and reduction of disulfide bonds can have deleterious effects on the structure, thus introducing a single cysteine residue via point mutation is a preferred approach to have a single tag attached to a precise protein location. Regardless of the above mentioned approaches, the classical *N*-hydroxysuccinimide (NHS) ester and other active esters remain the preferred functional group to attach fluorophore, chromophore, biotin and DNA to a protein via lysine residue^16,17^. The abundance of lysine residues in most proteins makes labeling easy, and it is a one-step reaction plus one-step purification. Nonetheless, due to the instability (hydrolysis) of the active ester, inconsistency of reaction efficiencies, and concerns of over-labeling affecting the protein’s function^18^, it remains a daunting task.

**Figure 1:**
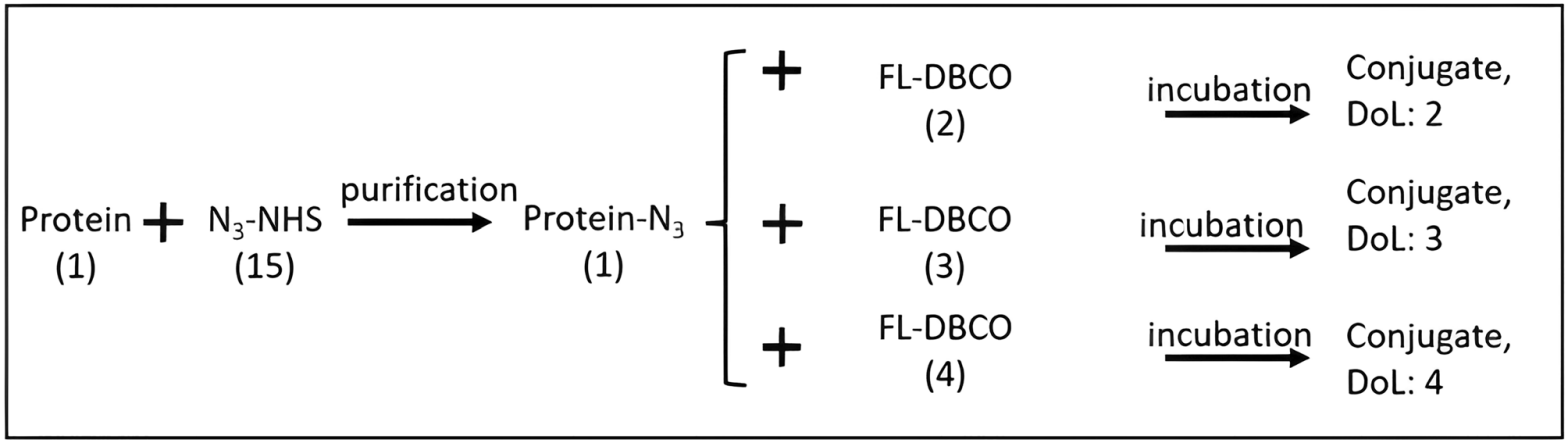
Scheme for click labeling with targeted aDoL. The number in parentheses represents the molar ratio in the reaction. Using 15X molar excess of N_3_-NHS will usually yield ~ 5 N_3_- per protein provided the protein has sufficient lysine residues. The average degree of labeling (aDoL) can be controlled with the reagents’ input molar ratio in the second reaction step. The tag can be DBCO, or other cyclooctynes complementary to N_3_-.

Here, we describe a labeling method that guarantees targeted aDoL using readily available copper-free click chemistry reagents. The attachment sites are stochastically targeted to the lysine residues on the protein surface, and the aDoL is the average number of tag (fluorophores) conjugated to each protein. It is a two-step reaction with one purification step in between (shown in Fig. 1). As a first step, the protein was activated with N_3_-NHS and excess N_3_-NHS was removed after the reaction. In the second step, the protein-N_3_ was mixed with fluorophore-DBCO at a molar ratio equivalent to the desired aDoL. After a 24-h incubation, the labeled protein is ready for use.

The success of the proposed labeling protocol relies on two conditions: (1) In the activation step, the average number of N_3_ attached to the protein should be higher than the desired aDoL, (2) the added fluorophore-DBCO must be fully consumed in the second step, thus making additional purification unnecessary. The protocol was tested on two protein systems, a small protein: apomyoglobin (apoMb, 17 kDa) and an antibody (150 kDa). They were both labeled with AZ488-DBCO, achieving aDoL of 2–4 for apoMb and aDoL of 2–8 for antibody. Chromatography and Fluorescence Correlation Spectroscopy (FCS) were used to monitor the labeling reaction, detect unreacted fluorophores, and characterize the brightness of the conjugates.

FCS measures the signal fluctuation of fluorescent molecules as they freely diffuse through a well-defined illumination volume (~ 1 fL). This elegant technique was introduced more than 30 years ago^19^, but was not frequently used until the mid-90’s when faster computers and advanced instruments became available. A comprehensive overview of the FCS basic principles and applications can be found elsewhere^20^. The calculated autocorrelation curve provides information on the diffusion rate of the fluorescent molecules. A smaller molecule (e.g., a free fluorophore) has a faster diffusion rate comparing to a fluorescently labeled protein and thus reflected in a right-shifted autocorrelation curve during the labeling reaction.

## Materials and methods

### Material

Azidoacetic acid NHS ester (N_3_-NHS) and all fluorophore-DBCO were purchased from Click Chemistry Tool (Scottsdale, AZ). AlexaFluor 488-SDP (AF488-SDP) and Zeba spin desalting columns were purchased from Thermo-Fisher Scientific (Waltham, WA). Cy5-NHS were purchased from GE healthcare (Buckinghamshire, U.K). NGAL, anti-NGAL antibodies, and anti-biotin antibody used in the study were produced in house by Abbott laboratory^21^ (Abbott Park, IL). Apomyoglobin was purchased from Sigma-Aldrich (St. Louis, MO.). Goat-anti-mouse antibody- Dylight 649 was purchased from Jackson ImmunoResearch Inc (Westgrove, PA). Chromlink Biotin was purchased from Vector Laboratories (Newark, CA).

### Methods

#### AlexaFluor 488-SDP labeling

The anti-NGAL Ab1 at 2 mg/mL in PBS buffer was mixed with eightfold molar excess of AF488-SDP ester. The reaction was carried out at 2–8 °C overnight. Excess AF488-SDP were removed by passing the sample twice through Zeba spin desalting columns.

#### Pre-activation for click labeling

1–2 mgs of N_3_-NHS was dissolved in DMSO to a final concentration of 10 mg/mL. The concentration of N_3_-NHS was determined by accurately weighing or measuring the hydrolyzed product, NHS, ε_260 nm_ = 9700/M/cm^22^. Both methods were used to determine the concentration of N_3_-NHS. The differences were within 5%. In the pre-activation step, a wide range of molar excess of N_3_-NHS (four to fivefold excess) were mixed with 2 mg/mL of antibody or 1.2 mg/mL of apomyoglobin to achieve various I.R. After a two hours incubation at room temperature, the unreacted N_3_-NHS were removed by passing the samples twice through Zeba spin desalting columns. Attachment of an azido group to the protein does not affect the absorption spectrum of the protein, thus the concentration of the Ab-N_3_ or apoMb-N_3_ is determined with Ab’s or apoMb’s extinction coefficient (Ab: ε_280_ = 217,500/M/cm, apoMb: ε_280_ = 15,900/M/cm).

#### Determination of I.R. for protein-N_3_

We have adopted two methods to determine the incorporation ratio (I.R.) of the N_3_- to protein. The Ab-N_3_ first reacted with excess molar of Cy5-DBCO (every N_3_ tag should has a Cy5-DBCO attached), the sample was injected onto analytical HPLC for analysis after 24 h incubation, the absorption spectrum at 8.8 min was used to determine the I.R. The following equation were used to calculate aDoL of Cy5 to Ab ([Cy5] = A_663_/255,000/M/cm; [Ab] = (A_280_ − 0.05 × A_663_)/217,500/M/cm; aDoL = [Cy5]/[Ab]), which would be a good estimate the I.R. of Ab-N_3_. The labeled ApoMbs were analyzed by TripleTOF® 5600 mass spectrometer (Sciex, Framingham, MA) coupled to an Eksigent MicroLC 200 HPLC (Sciex, Framingham, MA). The multiple charged states distribution of the protein samples was deconvoluted using reconstruct function available in Peakview software from AB Sciex. The average I.R. is calculated using formula: ∑(number of N_3_ attached to protein x peak intensity)/∑(peak intensity).

#### Labeling protein with fluorophores-DBCO

All fluorophore-DBCO stocks were dissolved in DMSO to a final concentration of 10 mg/mL, then diluted in PBS buffer to ~ 100 μM. The 2 mg/mL Ab-N_3_ (I.R. 7) was divided into small aliquots (50 μL), each reacted with 2× molar equivalent of AZ405-DBCO, AZ430-DBCO, AZ488-DBCO, AZ546-DBCO, AZ568-DBCO, AZ594-DBCO, or Cy5-DBCO. The products were analyzed on HPLC to determine reaction efficiencies.

#### Monitoring the click reaction by HPLC

The reaction kinetics of protein-N_3_ and AZ488-DBCO was monitored by analytical chromatography using the Agilent Chemstation. 50 μL of the mixture was injected onto the analytical SRT-C SEC 300 (HPLC column, Sepax) at various time (5 min–24 h). Changes in the elution profile reflect the progress of the reaction. In the experiment, where 2 mg/mL of Ab-N_3_ was reacted with nine different fluorophore-DBCOs, the reaction mixtures were injected onto the same column after a 24 ± 1.5-h incubation. There were 3 h of difference between the first and last sample injection. The detector was set at the absorption maximum of the corresponding fluorophore. The elution profiles were used to detect unreacted fluorophore-DBCO.

#### Fluorescence correlation spectroscopy (FCS)

FCS experiments were performed using fluorescence correlation spectrometer (ALBA, ISS, Champaign, IL) integrated with an inverted Nikon Eclipse TE300 fluorescence microscope (Nikon InsTech Co., Ltd., Kanagawa, Japan) and a Spectra-Physics Mai Tai Ti- Sapphire laser^23^. The system is calibrated with analytically prepared 20 nM AlexaFluor 488 before each use. All samples were diluted to 50–100 nM in 10 mM HEPES buffer (pH 7.4, containing 0.15 M NaCl, 3 mM EDTA, and 0.005% surfactant P20) and loaded in 384-well glass bottom plate for FCS measurement.

#### Impact of -N_3_ labeling on antibody binding activities

The antibodies used in the study are anti-NGAL antibody and anti-biotin antibody. Two independent protocols were used to compare the antibody’s binding activity. In one approach, serial titrations were performed on anti-NGAL Ab 1 with three different IRs (I.R. 7, 15, and 24) and unlabeled Ab 1. The Abs were diluted to 100 pM in 10 mM HEPES buffer. Each Ab was titrated with a serial of 2× diluted NGAL-Cy5 solution. After overnight incubation, the antibody or antibody-NGAL-Cy5 complex was captured by secondary antibody (goat-anti-mouse IgG Ab) coated microparticles, the anti-NGAL Abs used in the study are mouse antibodies. Fluorescence signal from NGAL-Cy5 captured on the microparticles were used to compare the binding activities of three Ab-N_3_ to unlabeled Ab.

In the second approach, four different anti-NGAL antibodies and one anti-biotin antibody were labeled with N_3_-NHS with different I.R.s ranging from 2 to 6. All labeled antibodies and their corresponding intact antibodies were diluted to 100 pM in 10 mM HEPES buffer. 100 μL of each sample first reacted with 5 μL 0.1% solid NGAL coated microparticles for 30 min and then 15 min of 50 μL 30 nM Goat-anti-Mouse Antibody F(ab’)_2_-Dylight649. The microparticles were washed after each binding reaction step, and then imaged on an Olympus epifluorescence microscope (instrument setup and image analysis were previous described^24,25^). The biotin-NGAL coated microparticles were prepared by mixing 10 μL 15 μM biotin-NGAL, to 1 mL 0.1% solid streptavidin microparticles. Unbound biotin-NGAL were washed away from microparticle solution before each experiment using plate washer. The streptavidin coated microparticles were prepared by coupling 0.1 mg/mL streptavidin to 5μm paramagnetic carboxyl particles (Polymer Lab, Palo Alto, CA) kept at 1% (w/w) solids in the presence of 0.15 mg/mL EDAC (1-ethyl-3-(3-imethylaminpropyl) carbodiimide, hydrochloride. Streptavidin microparticles without the coating of biotin-NGAL was used as a negative control to ensure the measured signals were specific binding between the antibody and its corresponding antigen.

## Results

Multiple labeling conditions and labeling products were described in this study, the following nomenclatures were used for clarity: protein-15×-N_3_-2×-AZ488, indicating the protein first reacts with 15× molar equivalent of N_3_-NHS, and then 2× molar equivalent of AZ488-DBCO. The number of N_3_-attached to the protein is referred to as the incorporation ratio (I.R.). The number of final fluorophores attached to the protein is referred as the average degree of labeling (aDoL). The intermediate product is referred as Protein-N_3_, the final product with fluorophores will be termed as the Conjugate.

### Labeling antibody with AZ488-DBCO at an aDoL of 2

**Figure 2:**
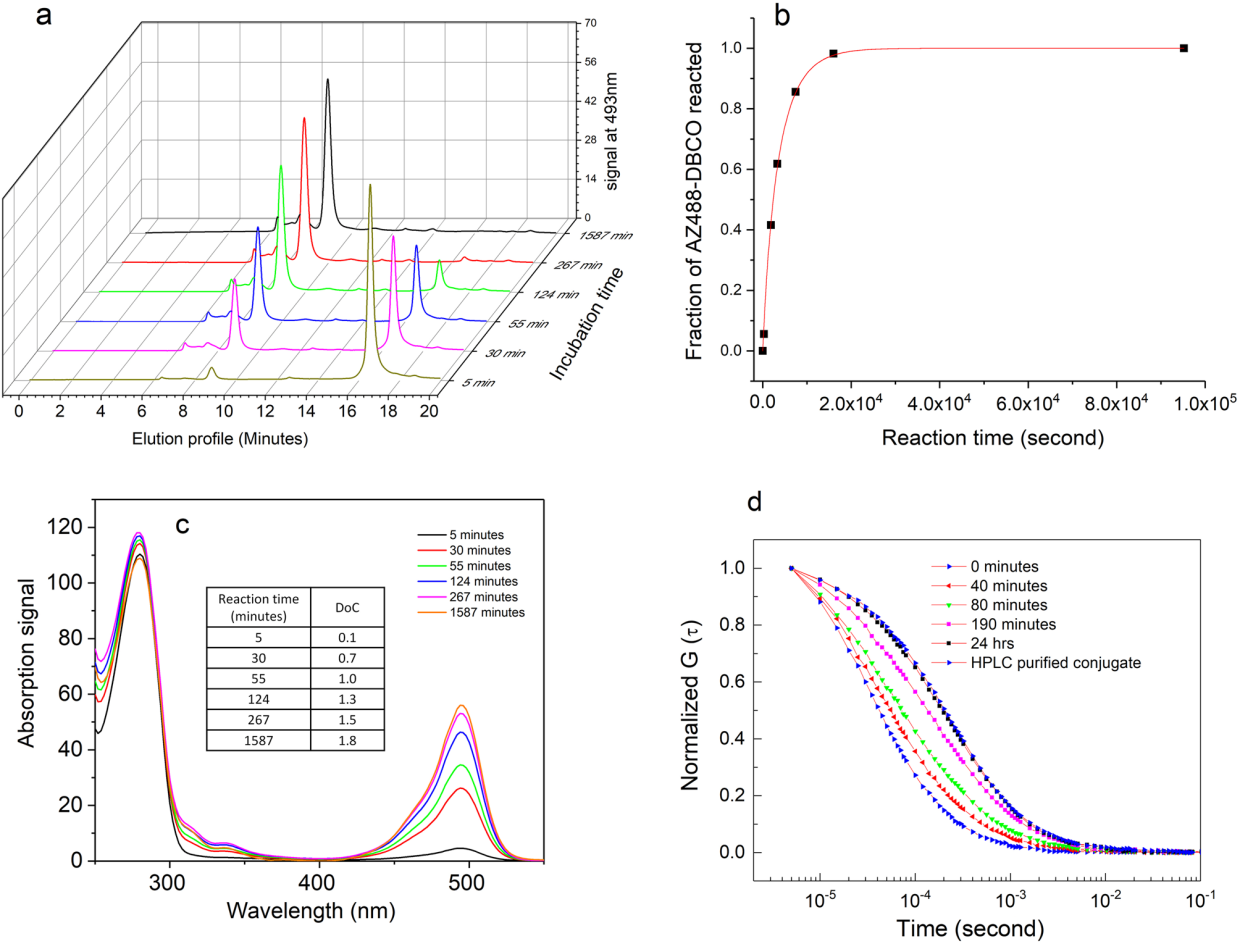
(**a**) Chromatograms of Ab-16×-N_3_ reacting with 2× AZ488-DBCO as a function of time, monitored at 493 nm. Conjugate Ab-16×-N_3_-2×-AZ488 elutes at 8.8 min, free AZ488-DBCO elutes at 16.5 min. (**b**) Reaction kinetic curves extracted from the chromatograms with a fitted K value of 4.31/M/s. (**c**) Absorption spectra of the conjugates from various reaction time measured by the PDA on the HPLC instrument. The legend shows the reaction time. (**d**) Autocorrelation curves of AZ488-DBCO reacting with Ab-N_3_ as a function of time. The curves shift to the right as more AZ488-DBCO are covalently attached to Ab-N_3_.

In the first example, the antibody was activated with 16× molar excess of N_3_-NHS, achieving an I.R. of 5.6. Then a 2:1 molar ratio of AZ488-DBCO and Ab-16×-N_3_ were mixed to achieve an aDoL of 2. The reaction mixture (Ab-16×-N_3_, AZ488-DBCO) was injected onto the analytical HPLC column at various times to monitor the reaction progress and the product Ab-AZ488 was collected for further analysis. Figure 2a shows examples of chromatograms monitored at 493 nm over time. The conjugate Ab-16×-N_3_-2×-AZ488 elutes at 8.8 min, while the free AZ488-DBCO elutes at 16.5 min. At the beginning of the reaction (5 min), there was only trace amount of the conjugate, and most fluorophores were in the form of free AZ488-DBCO. At 55 min, more than 50% of the fluorophores were attached to the antibody, and 267 min, there was only trace amount of free AZ488-DBCO. By 24 h, no free AZ488-DBCO was eluted off the column, indicating all AZ488-DBCO were bound to Ab-N_3_, thus making it safe to assume we have achieved the targeted aDoL of 2. Figure 2b shows the kinetic trace of the reaction extracted from the elution peak value at 8.8 min. The kinetic trace was then fit using Eq. (2) derived from the integrated rate equation for second order reaction (Eq. 1).

1 $$K=\frac{1}{t\times \left({[A]}_{0}-{[B]}_{0}\right)}{\text{Ln}} \left[\frac{{[B]}_{0}\left({[A]}_{0}-x\right)}{{[A]}_{0}\left({[B]}_{0}-x\right)}\right],$$

2 $$\text{Fraction of AZ488-DBCO reacted}=\frac{x}{{[B]}_{0}}=\frac{{\left[A\right]}_{0}\times \left({\text{exp}}^{Kt\left({\left[A\right]}_{0}-{\left[B\right]}_{0}\right)}-1\right)}{{\left[A\right]}_{0}\times {\text{exp}}^{Kt\left({\left[A\right]}_{0}-{\left[B\right]}_{0}\right)}-{\left[B\right]}_{0}},$$

where [A]_0_ and [B]_0_ are the initial concentrations of N_3_-DBCO, and AZ488-DBCO, respectively; *t* is the reaction time in seconds, *x* is the concentration of AZ488-DBCO attached to the protein, and K is the reaction rate constant. The fitted K is 4.31 ± 0.13/M/s.

We theoretically and experimentally confirmed that the click reaction was 100% complete after 24-h incubation provided the DBCO-tag and N_3_-tag were at minimal concentration of 10 μM and 25 μM, respectively. However, we did observe changes of the reaction rate constant or incomplete reaction on the second step depending on the size of the payload and targeted DoL, which might require longer incubation or target at a lower aDoL (see Supplementary section).

Figure 2c shows the absorption spectra of the conjugates (from various incubation lengths) at the 8.8 min elution point. The increased 495 nm peak indicates more AZ488 were attaching to the antibody over time. The inset table lists the aDoL calculated from each absorption spectrum using the equation described below. After 24 h of incubation, the conjugate achieved an aDoL of 1.8, which is close to the target value of 2.

$$\left[AZ488\right]=\frac{{A}_{495}}{\mathrm{70,000}}; \left[\mathrm{Ab}\right]=\frac{{A}_{280}-0.147\times {A}_{495}}{\mathrm{217,500}}; a\mathrm{DoL}=\frac{\left[\mathrm{AZ}488\right]}{\left[\mathrm{Ab}\right]}.$$

In parallel, FCS measurements were used to follow the progress of the reaction as well. The reaction mixtures were measured on the microscope without any purification step. Figure 2d shows the autocorrelation curves of the reaction mixture at various reaction time. The right shifted autocorrelation curves indicate more AZ488-DBCO are attached to the protein over time. At 24 h, the autocorrelation curve of the non-purified reaction mixture is superimposed with that of the HPLC purified conjugate, indicating all AZ488-DBCO had reacted with Ab-N_3_. Thus, we have used two independent methods to confirm the 100% completion of the labeling reaction.

### Microscale and parallel labeling

**Figure 3:**
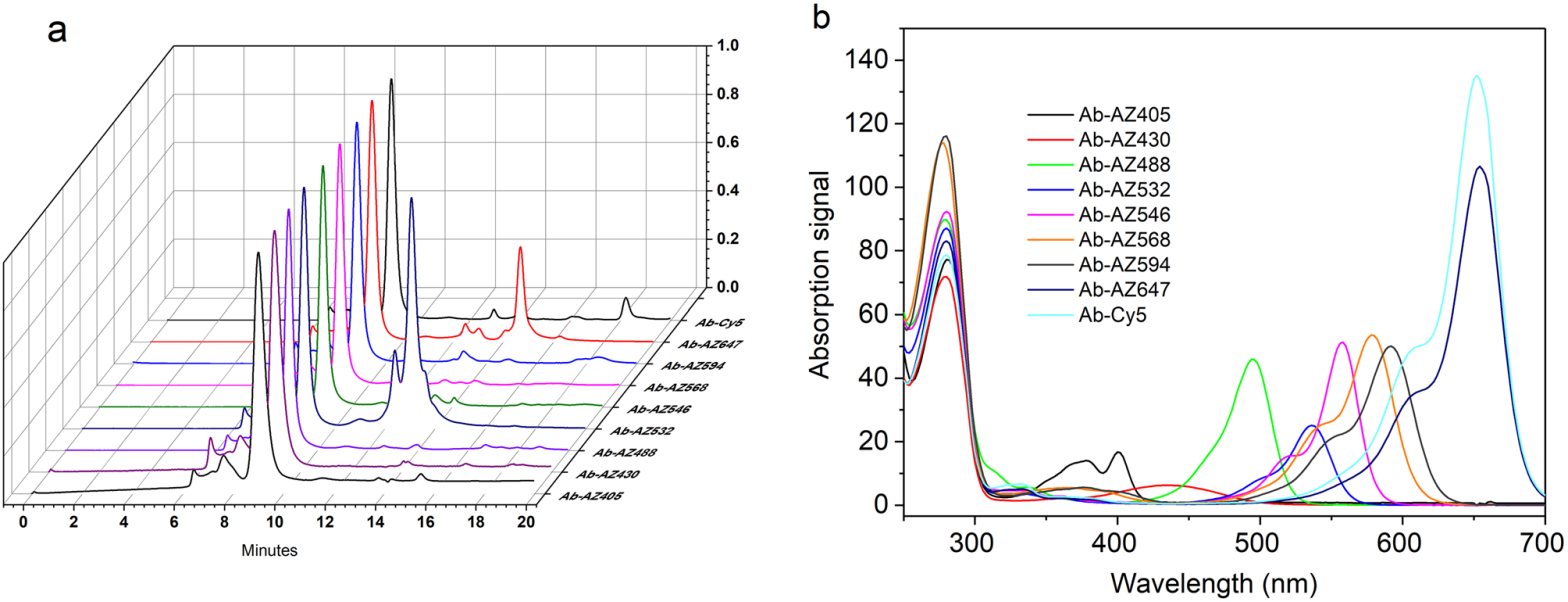
(**a**) HPLC elution profile (normalized) of the conjugates (Ab-17X-N_3_-fluorophore) after ~ 24-h’s incubation, each monitored at corresponding fluorophore wavelength. (**b**) Absorption spectra of the conjugates at 8.8 min elution point.

0.5 mg of Ab-17×-N_3_ was divided into 9 aliquots and each reacted with 2× molar equivalents of AZ405-DBCO, AZ430-DBCO, AZ488-DBCO, AZ546-DBCO, AZ568-DBCO, AZ594-DBCO and Cy5-DBCO. All products were loaded onto the analytical HPLC column after ~ 24 h of incubation and were monitored at maximum absorption wavelength of each corresponding fluorophore. Unreacted residuals were found only in samples AZ532-DBCO and AZ647-DBCO. The rest all fully reacted with the protein (Fig. 3). We were unsure the cause of the incomplete reaction for AZ532-DBCO and AZ647-DBCO, but those two compounds should be avoided. The purpose of this experiment was to demonstrate that, after the activation step, the protein-N_3_ can be conjugated with fluorophores, biotin or other small payloads with DBCO tag in parallel at microscales without additional need of purification, but one should confirm 100% reactivity of the DBCO-reagent with N_3_- tag during initial testing.

### Comparison of the stability of attached fluorophore via click labeling or direct active ester-fluorophore labeling

Previously, when labeling proteins with AF488-SDP or AF488-NHS, free fluorophores were always detected in the conjugate solution after storing for only a few days. It has been suggested that the fluorophores were non-covalently attached to protein during the labeling reaction, and then would dissociate from the protein slowly over time, resulting in free fluorophore present in the purified protein conjugate. We feel N_3_-NHS should be less of a problem in that respect. The conjugated Ab-AF488 (direct attached through SDP ester functional group) and Ab-17×-N3-2×-AZ488 were prepared on the same day and measured on day 1 and day 100 using the FCS instrument. After 100 days at 2–8 °C, 15% of free AF488 was detected in the Ab-SDP-AF488 conjugate, while the conjugate labeled via SPAAC reaction had no free fluorophores detected (see S Fig. 5). This direct comparison experiment indicated that our click labeling approach can produce more stable conjugate, which can be explained by non-covalently bound fluorophore-DBCO (if any) can always react with protein-N_3_ over time).

### Labeling antibodies over a wide range of targeted aDoLs

**Figure 4:**
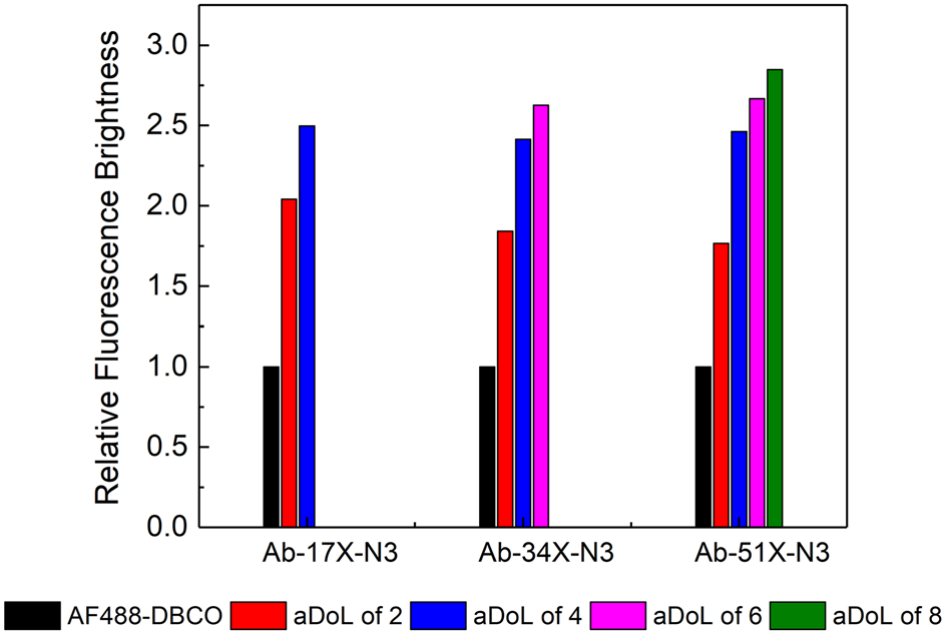
Fluorescence brightness of the conjugates calculated from FCS measurements. At aDoL of 2, the brightness of the conjugate is twice of single fluorophore AZ488-DBCO, there are incremental increase of brightness with higher aDoL, but does not follow the linear trend.

The antibody was first reacted with 17×, 34× and 51× molar excess of N_3_-NHS, and achieved an I.R. of 7, 15, and 24 respectively. The protein-N_3_ was then reacted with AZ488-DBCO at molar ratios of 2, 4, 6, and 8. After 24-h incubation, the samples were diluted for fluorescence correlation spectroscopy (FCS) measurements without purification. As expected, AZ488-DBCO reacted 100% with Ab-17×-N_3_ (I.R. 7) at aDoL target of 2 and 4, but trace amounts of AZ488-DBCO was detected for Ab-17×-N_3_ (I.R. 7) at aDoL target of 6 and 8. On the other hand, Ab-34×-N_3_ (I.R. 15) could accommodate up to 6 AZ488 per molecule, and Ab-51×-N_3_ (I.R. 24) could accommodate up to 8 AZ488 per molecule (see S Fig. 4). A similar reaction protocol was performed on apoMb and achieved the targeted aDoL as expected (see S Fig. 3). This study suggests the I.R. of the protein-N_3_ should be twice of the targeted aDoL to ensure the completion of the click reaction. However, it is known that the fluorescence brightness of labeled protein is not always linearly proportional to the number of fluorophores attached to it due to self-quenching, or sometimes referred to as concentration quenching^26,27^. We observed the expected self-quenching phenomena of the labeled protein with the FCS measurements. Figure 4 shows at aDoL of 2, the brightness of the conjugate is twice that of a single fluorophore AZ488-DBCO. There are incremental increases of brightness with higher aDoL, but it does not follow the linear trend. At aDoL of 8, the brightness of the conjugate is merely 3 times the brightness of fluorophore AZ488. Considering the potentially negative impact of extensive labeling to the protein’s structure and function, and the minimal gain in brightness with higher aDoL^28^, it is important to emphasize that the aDoL should be kept low. Assuming the number of fluorophores per antibody follows a Poisson distribution^18,29^, even at aDoL of ~ 2, there will be ~ 18% of the protein with 3 labels, and ~ 15% of the protein with 4 and above labels.

### Determination of I.R. and labeling efficiency for protein -N_3_

The functional tag, N_3_- does not absorb at 260 nm nor at 280 nm, thus attachment of N_3_- to the protein cannot be confirmed by absorption spectrum. Proteins with different I.R has very similar absorption spectra (see S Fig. 1). We have adopted two methods to determine the incorporation ratio of the N_3_- to protein. In one approach, the protein-N_3_ reacts with excess molar of Cy5-DBCO, when every N_3_ tag has a Cy5-DBCO attached, the aDoL of Cy5 to protein-N_3_ should reflect the I.R. of Ab-N_3_ and the aDoL can be determined by absorption spectra. This approach is more suitable for larger protein (e.g., Ab), which has a higher extinction coefficient and sufficient space to accommodate the fluorophores. Supplementary Table 1 listed the peak value and calculated aDoL of Ab-N_3_-DBCO-Cy5. An alternative approach is to use ESI–MS and directly resolve the number of N_3_- by the mass increase of the protein. This is more suited for smaller size protein without glycosylation (e.g., ApoMb). ESI–MS Spectra of apoMb-N_3_ are included in Supplementary section (S Fig. 2). Table 1 lists the I.R. of Ab-N_3_ and apoMb-N_3_, both methods show the reaction efficiency falls in the range of 25–47%. Excluding the over-labeled cases, the reaction efficiencies are at ~ 30% with the I.R. levels below 5.

**Table 1:** Labeling antibody and apoMb with N_3_-NHS at various ratios.

Sample	I.R	Reaction efficiency of N_3_-NHS to protein (%)
ApoMb-15×-N_3_	4.6	31
ApoMb-30×-N_3_	7.3	25
Ab-4×-N_3_	1.3	33
Ab-8×-N_3_	2.5	31
Ab-12×-N_3_	4.2	35
Ab-16×-N_3_	5.6	35
Ab-17×-N_3_	7	40
Ab-34×-N_3_	15	44
Ab-51×-N_3_	24	47

### Impact of N_3_ labeling to antibody activity

**Figure 5:**
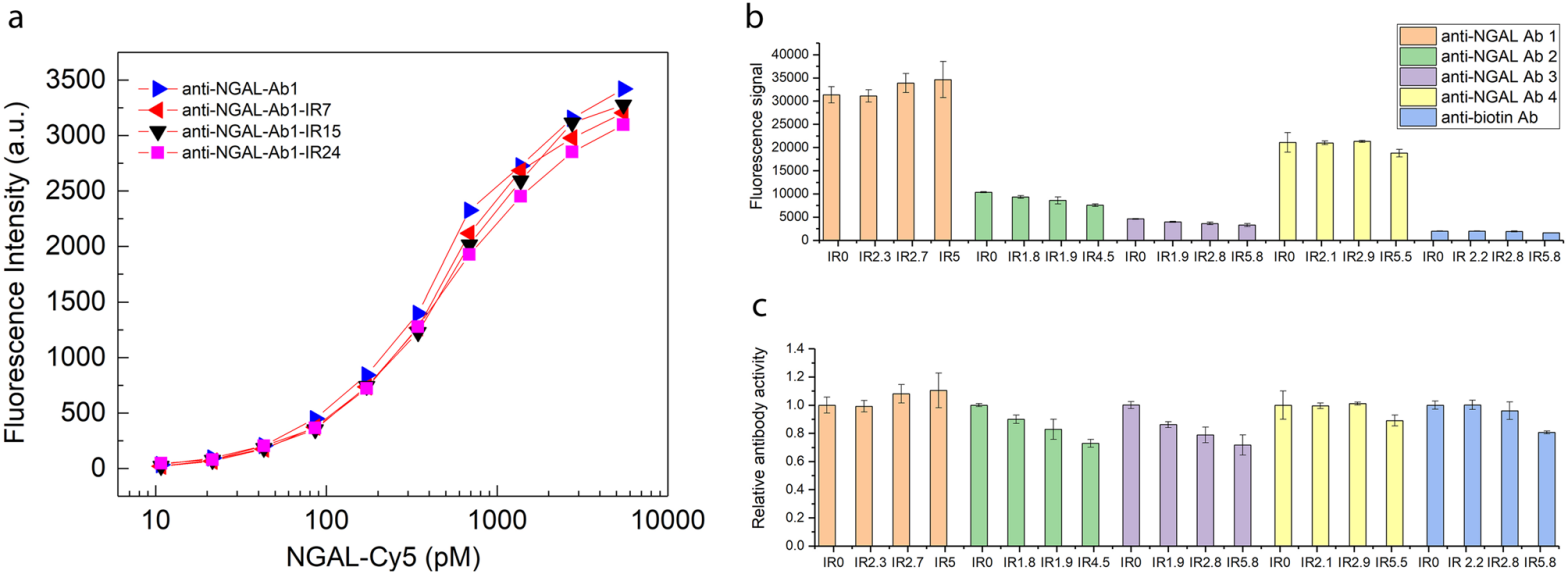
(**a**) Binding titration of Ab1-N_3_ and its antigen, NGAL-Cy5. Unlabeled Ab1 and Ab1-N_3_ were kept at 100 pM, while the concentration of NGAL-Cy5 varied from 10 pM to 5.5 nM. All three Ab-N_3_ have similar binding activity as the native Ab, indicating minimal impact of the N_3_ attachment to Ab. (**b**) Fluorescence signal of antibody captured by corresponding antigen. (**c**) Relative antibody activity of the N_3_ labeled antibodies to its corresponding intact antibody.

Two independent protocols were used to compare the antibody’s binding activity upon -N_3_ modification. In the first approach, binding titration curves were performed on anti-NGAL Ab1 at IRs of 0, 7, 15 and 24. Each binding curve has 10 data points. All binding curves are superimposable, indicating no impact of N_3_ modification to the Antibody’s function (Fig. 5a). In the second approach, we adopted a simpler method to evaluate more antibodies with different IRs (see “Materials and methods” section). All reagent (NGAL coated microparticles, goat-anti-mouse Ab-Dylight 649) concentrations and reaction conditions were kept same. Figure 5b shows the fluorescence signal of the original and N_3_ labeled antibodies when binding to their corresponding antigen. The five antibodies have inherent different affinity toward its targeted antigen, which are reflected in the varied signal levels for each antibody. However, the signal variation from the same antibody labeled with different I.R. reflects the impact of labeling modification. Figure 5c shows normalized signal of labeled Abs to intact Ab. Anti-NGAL Ab1 and Ab4 showed the attachment of N_3_ have no negative impact on the proteins’ function, while anti-NGAL Ab2, Ab3 and anti-biotin Ab showed up to 30% binding activity loss at higher IRs. And there is an increase trend of functional loss with increased IR. The negative control experiment (Streptavidin coated microparticles) indicated all antibodies bind to NGAL or biotin specifically, the non-specific-binding signal at 100 pM Ab level is negligible.

## Discussion

The labeling technique presented here is straightforward and precise. We are not aware of any existing labeling method which has the same features without performing chemo or site-selective labeling. As mentioned in the introduction, the major drawback of using active ester-fluorophore for protein conjugation is the inability to precisely control aDoL due to the instability of active ester-fluorophore and unpredictable reaction efficiency. Not all active ester tags are created equal. Heavily sulfonated dyes, such as the Alexa Fluor® dyes, DyLight® dyes and IRDye® are particularly hygroscopic, further worsening the hydrolysis of active esters. In one incidence, we detected 37% of hydrolyzed Alexa Fluor® 488-NHS in a freshly opened vial by LC–MS (see S Fig. 5). The manufacture manual also acknowledged the % of active reagent are usually > 50%, but can be as low as 30–40%. With unknown level of hydrolysis, and the bulky size of the fluorophores, it is difficult to predict the reaction efficiency. Based on our experience, the labeling efficiency of AF488-NHS or AF488-SDP to protein can vary from 5 to 50%. Whereas N_3_-NHS is small, the intact N_3_-NHS and hydrolyzed N_3_-NHS have different absorption spectra, which could be used to check the integrity of the labeling reagent^22^. We tested various labeling ratios of N_3_-NHS to apoMb and antibody, the reaction efficiencies were at ~ 30% for both proteins with the I.R. below 5. We don’t expect 30% reaction efficiency for every protein and every lot of N_3_-NHS, but it does show consistency and reproducibility. Furthermore, in a case of over-labeling, each additional N_3_- attachment is only 83 Dalton, it would have less impact to the protein’s function comparing to a bulky fluorophore.

The utility of this method was further demonstrated by the parallel labeling of 9 different fluorophores to the same protein-N_3_ in a batch mode approach. After the activation step, the protein-N_3_ can be conjugated with fluorophores, biotin or other small payloads with DBCO tag in parallel at microscales without additional need of purification. In a recently published paper^30^, the author tried to evaluate the effect of antibody biotinylation on an immunoassay using various linker lengths and various of aDoL on three antibodies. In total, 150 labeling conditions were performed, which requires 150 independent purifications. If our approach would have been used, the three antibodies would first be activated with N_3_-NHS, after purification, each antibody could then be divided into 50 aliquots and reacted with various ratios of the 5 linkers, and the resulting product could be directly used without further purification. The total number of purifications would have been reduced from 150 to 3.

As shown in the “Result” section, return of benefit diminishes with higher aDoL for fluorophore labeling due to fluorescence self-quenching and possible impact on protein’s function. We have tested five different antibodies with different I.Rs and compared the N_3_ impact on protein’s function. As expected, some antibodies maintained its binding activity while others lost 30% of its ability binding to its antigen at higher I.Rs. Although some protein can accommodate over 20 N_3_ tags, but every protein is different, it is important to confirm the property of the protein after any modification. We recommend introducing 3–5 -N_3_ attachment to each protein, which is enough to accommodate 2 DBCO-fluorophores in the final reaction step. It is also possible to use TCEP to reduce the unreacted azido group on the protein back to amine after reacting with DBCO, however, it is important to confirm that the TCEP did not break the disulfide bond of the protein.

Overall, we have performed extensive studies to demonstrate that our approach provides a simple way to parallel label proteins at low quantity with well controlled aDoL.

## Supplementary Information

Supplementary Information.

## Data Availability

The data are available from the corresponding author on reasonable request.

## Abbreviations

aDoL: Average degree of labeling
I.R.: Incorporation ratio
FCS: Fluorescence correlation spectroscopy
