# Supplementary material for: A method for parallel microscale protein labeling and precise control over the average degree of labeling (aDoL)
Source: Sci Rep. 2023 Jun 2;13:8961. doi: 10.1038/s41598-023-36163-8 (PMC10238424; doi:10.1038/s41598-023-36163-8)
Supplement: Supplementary file 1 — Supplementary Information. [file 41598_2023_36163_MOESM1_ESM.docx]

A method for parallel microscale protein labeling and precise control over the average degree of labeling (aDoL)

Qiaoqiao Ruan, Cheng Zhao

Applied Research and Technology, Abbott Diagnostics Division, Abbott Laboratories, Abbott Park, IL 60064

Corresponding author: [Qiaoqiao.ruan@abbott.com](mailto:Qiaoqiao.ruan@abbott.com),

**I.R. Determination for protein-N_3_:**

The functional tag, N_3_- does not absorb at 260 nm nor at 280 nm, thus attachment of N_3_- to the protein cannot be confirmed by absorption spectrum. Proteins with different I.R has very similar absorption spectra (See S Figure 1). For characterization purposes, we have adopted two methods to determine the incorporation ratio of the N_3_- to protein. In one approach, the protein-N_3_ was reacted with excess molar of Cy5-DBCO and after 24 hours, the samples were injected into analytical HPLC for analysis. The absorption spectra at 8.8 min elution time point can be used to determine the I.R. This approach is more suitable for larger proteins (e.g., Ab), which has higher extinction coefficient and sufficient room to accommodate the fluorophores. The following equation were used to calculate DoL of Cy5 to Ab ([Cy5]=A_663_/255,000M^-1^cm^-1^; [Ab]=(A_280_-0.05*A_663_)/217,500M^-1^cm^-1^; DoL=[Cy5]/[Ab] ), and the DoL of Cy5 to Ab would be a good estimate the I.R. of Ab-N_3_.

S Figure 1: Absorption spectra of Ab-N_3_ at various I.R. N_3_- has no characteristic absorption feature, thus the I.R of Ab-N_3_ cannot be determined by absorption spectra.

S Table 1: Absorption peak value of Ab-N_3_-DBCO-Cy5, the calculated I.R. and the N_3_-NHS reaction efficiency.

| Sample | A650 | Conc (μM) | A280 | Cor A280 | mM | IR | Reaction efficiency |
| --- | --- | --- | --- | --- | --- | --- | --- |
| Ab-16x-N_3_-Cy5 | 0.335 | 1.34 | 0.082 | 0.05185 | 0.24 | 5.62 | 0.35 |
| Ab-12x-N_3_-Cy5 | 0.287 | 1.15 | 0.086 | 0.06017 | 0.28 | 4.15 | 0.35 |
| Ab-8x-N_3_-Cy5 | 0.252 | 1.01 | 0.111 | 0.08832 | 0.41 | 2.48 | 0.31 |
| Ab-4x-N_3_-Cy5 | 0.152 | 0.61 | 0.113 | 0.09932 | 0.46 | 1.33 | 0.33 |

An alternative approach is to calculate the average incorporation ratio based on ESI-MS spectrum. It is more suited for smaller size protein without glycosylation (e.g. ApoMb). The labeled ApoMbs were analyzed by TripleTOF® 5600 mass spectrometer (Sciex, Framingham, MA) coupled to an Eksigent MicroLC 200 HPLC (Sciex, Framingham, MA)., the multiple charged states distribution of the protein samples was deconvoluted using reconstruct function available in Peakview software from AB Sciex.. The average I.R. is calculated using formula:

∑(number of N_3_ attached to protein x peak intensity) ⁄ ∑(peak intensity)


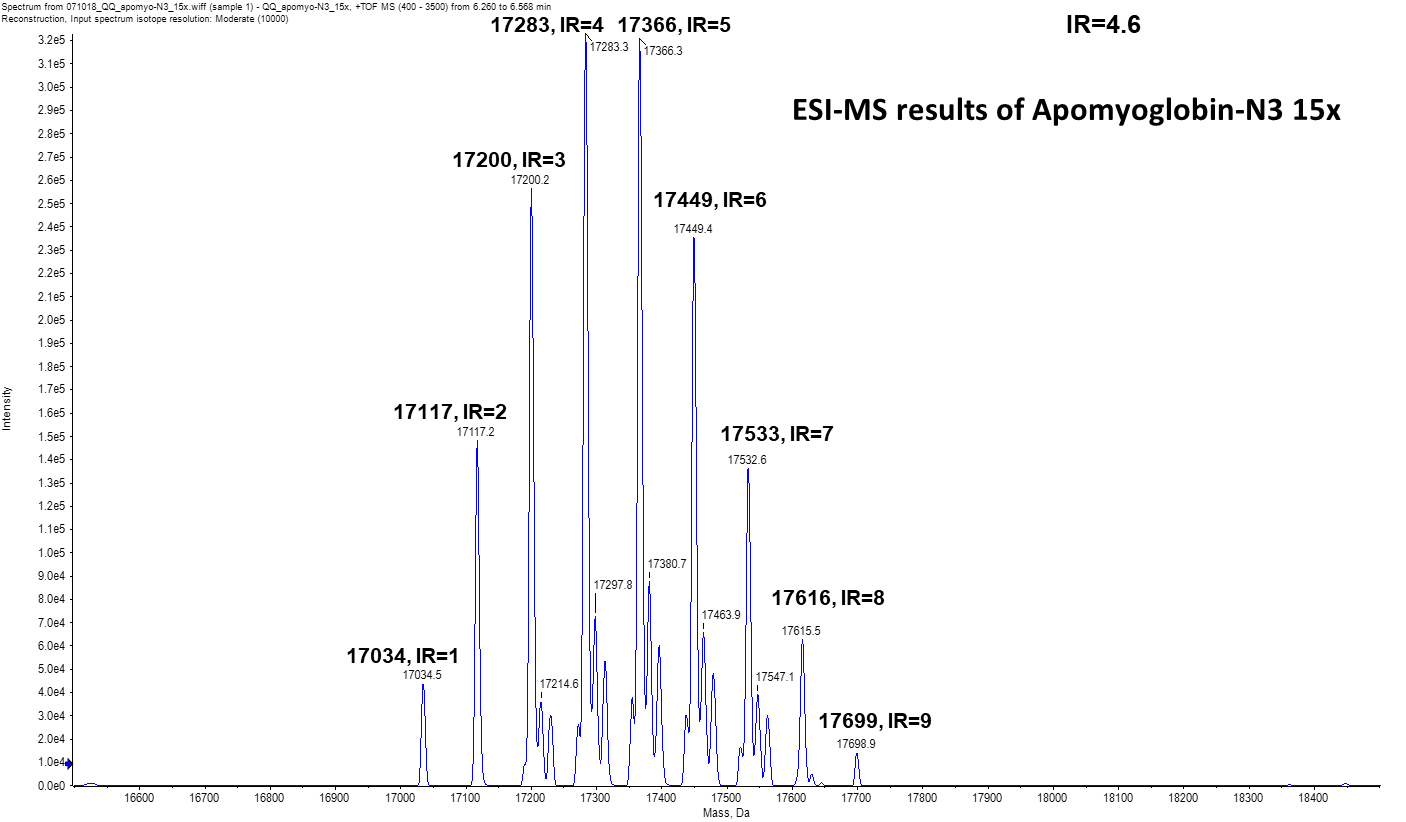


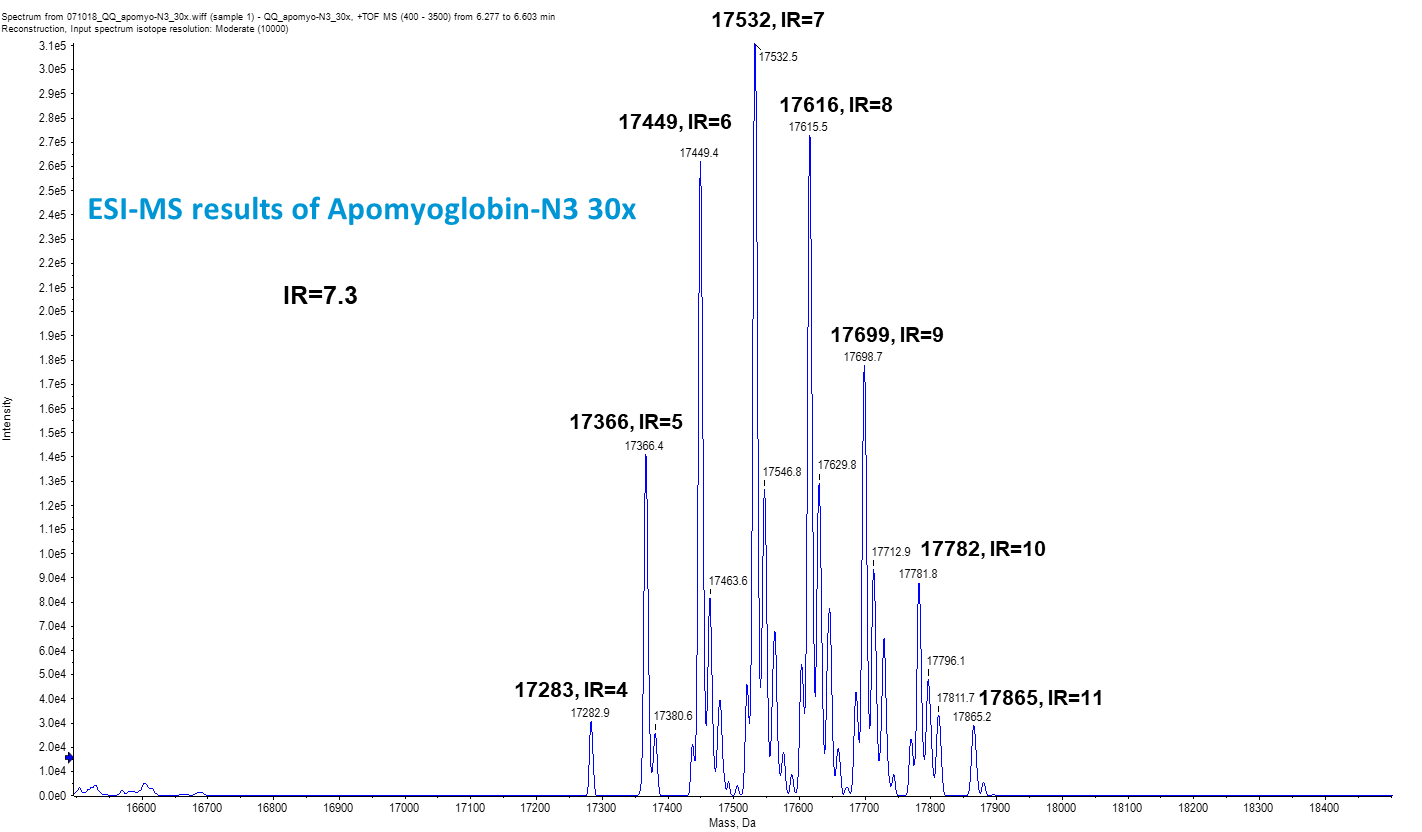


S Figure 2: ESI-MS results of Apomyoglobin-N_3_

| Sample | I.R. determined by ESI-MS | Reaction efficiency of  N_3_-NHS to protein |
| --- | --- | --- |
| ApoMb-15X-N_3_ | 4.6 | 0.31 |
| ApoMb-30X-N_3_ | 7.3 | 0.24 |

**Labeling ApoMb with AZ488 at desired DoC**

ApoMb is a 17 kDa protein and functions as a good representative of other small proteins. After reacting with various amount of the N_3_-NHS tag, 2X, 3X and 4X molar equivalent of AZ488-DBCO were added to the N_3_-ApoMb. After a 24-hour incubation, the products were run on the analytical HPLC column and monitored at 490 nm. The conjugate comes off the column at 11.1 minutes elution point, while the free AZ488-DBCO comes off the column at 16.5 minutes. As a small amount of free AZ488-DBCO (7%) was detected only in sample ApoMyb-16X-N_3_-4X-AZ488 (I.R.=4.6), it is safe to assume all the rest of the conjugates achieved the desired aDoL (i.e., 2,3,4).


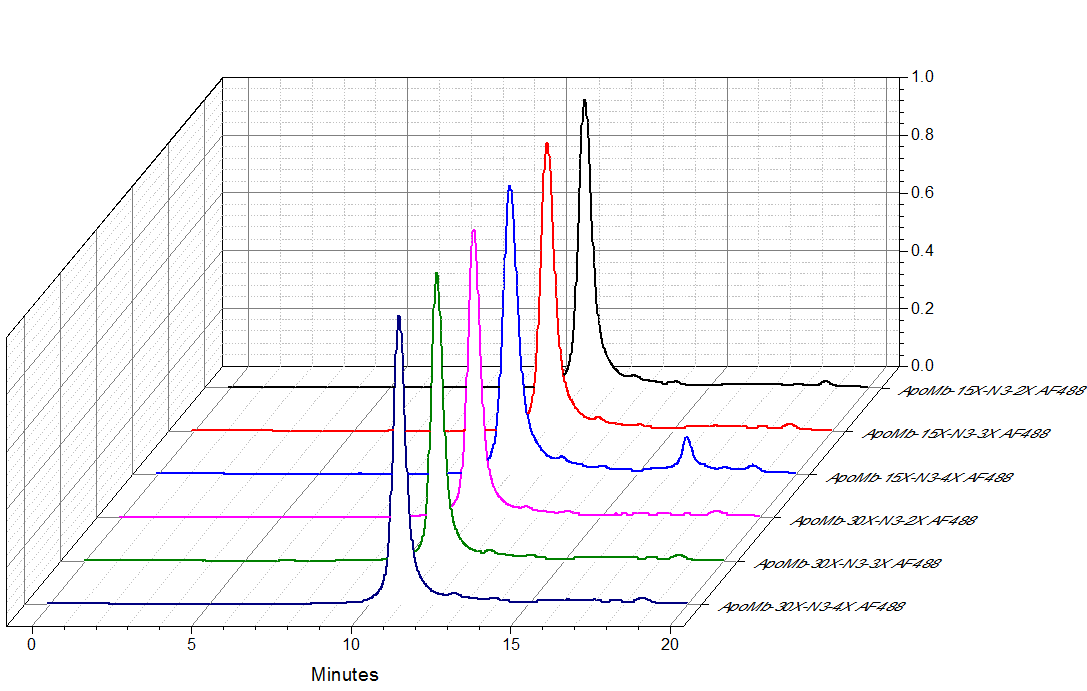


S Figure 3: HPLC analysis of the conjugates (ApoMb-N_3_-AZ488) after 24 hours incubations.

**Fluorescence correlation spectroscopy (FCS):**


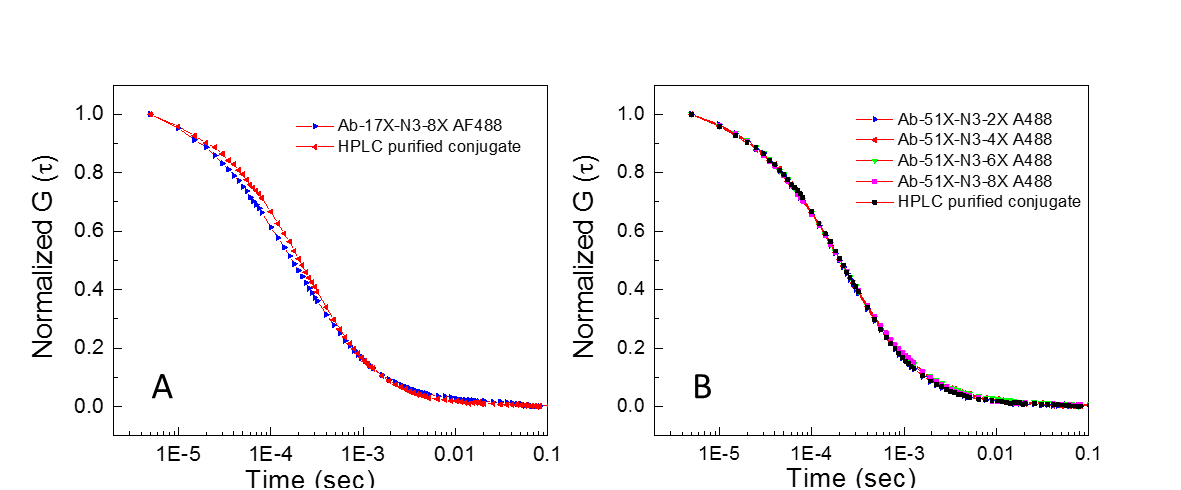


S Figure 4: Examples of autocorrelation curves of the conjugate labeled at various DoL without purification. A). The autocorrelation curves of the HPLC purified conjugate does not superimpose with Ab-17X-N_3_-8X-AZ488, indicating the presence of unreacted AZ488-DBCO. The Ab-17X-N_3_ has an I.R of 7, and did not have sufficient N_3_ to react with an 8-fold concentration of AZ488-DBCO. B). The autocorrelation curves of the HPLC purified conjugate superimpose with all Ab-51X-N_3_-xx-AZ488 conjugates, indicating complete reaction of AZ488-DBCO with Ab-51X-N_3_. The Ab-51X-N_3_ has an I.R. of 24, and had sufficient N_3_ to react with 8 fold of AZ488-DBCO.

S Figure 5:

Stability of the conjugates. Top). Autocorrelation curves of Ab-17X-N_3_-2X-AZ488 (SPAAC) on day 1 and day 100. Bottom). Autocorrelation curves of Ab-SDP-AF488 on day 1 and day 100. Free AF488 were detected on day 100.

**Hydrolysis of AF488-NHS**


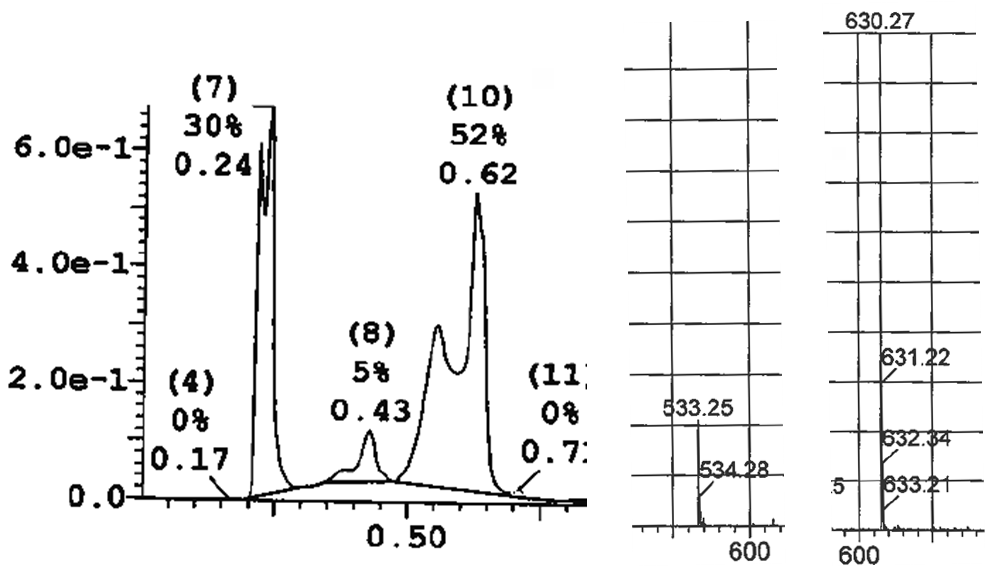


S Figure 6: The purity of AF488-NHS from a new vial was checked on UPLC/MS, peak (7) corresponds to the hydrolyzed AF488-NHS with a molecular mass of 533.25. Peak (10) corresponds to the intact AF488-NHS with a molecular mass of 630.27. % of the hydrolyzed AF488 can be calculated using the peak area, which is 37%.

**Reaction rate of DBCO and N_3_ can be affected by the size of payload**

**Label oligonucleotide with Cy5-DBCO:**

14 μM of a 38mer oligonucleotide (IDT, ref 156298577) with N_3_- modification reacted with 10.8 μM Cy5-DBCO. Progress of the reaction was followed by injecting the sample onto HPLC.

**
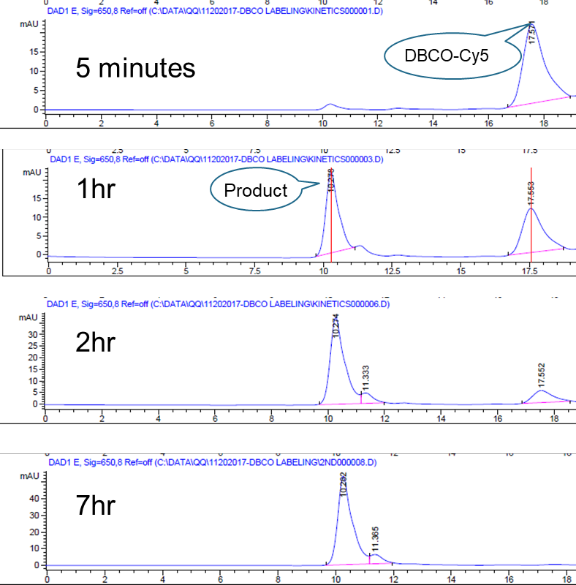
**

S Figure 7: A) Examples of the reaction mixture HPLC elution profile over time, reflecting the progress of the reaction. B) The kinetic trace was extracted from the peak value at 10.2 minutes. The fitted reaction rate constant is 25 M^-1^s^-1^.

**Conjugate oligonucleotide to Ab:**

The antibody was first reacted with 20-fold excess of DBCO-NHS, and an IR of 10 was achieved. Then a 38mer oligonucleotide (IDT, ref 156298577) IDT with N_3_- modification reacted with Ab-20x-DBCO at various Ab: oligo ratios (1:1, 1:2, 1:4, and 1:6). Progress of the reaction was followed by injecting the sample onto HPLC. At the 1:1 ratio, the reaction was >90% complete at 4 hours, and 100% complete at 18hrs. The calculated reaction rate constant is 4M^-1^s^-1^, close to the protein-N_3_ and fluorophore- DBCO reaction rate. However, at the 1: 2 ratio, the reaction rate was significantly reduced to 2.7 M^-1^s^-1^, and the reaction was not able to reach 100% completion, indicating that the size of the payload could affect the reaction rate and reaction efficiency See figure below.


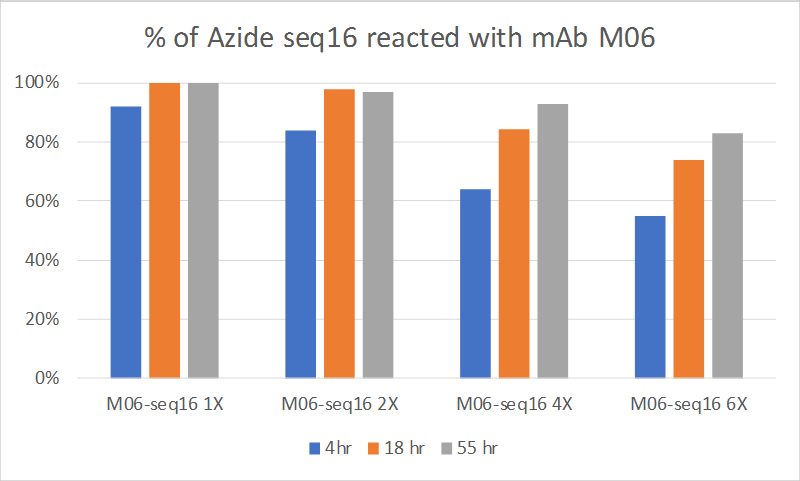


S Figure 8: Percent of reaction completion at 4, 18 and 55 hrs of incubation time and with different Ab: Oligo molar ratios (1:1, 1:2, 1:4, 1:6) The I.R of Ab-N_3_ is 10.
